# Grayken Lessons: a patient who developed opioid use disorder after traumatic brain injury

**DOI:** 10.1186/s13722-024-00525-y

**Published:** 2024-12-18

**Authors:** Gabriela Reed, Hansel Lugo, Rachel Sayko Adams, Alexander Y. Walley

**Affiliations:** 1 Department of Medicine, Division of General Medicine and Clinical Epidemiology, University of North Carolina School of Medicine, Chapel Hill, NC USA; 2 Massachusetts General Hospital, Boston, MA USA; 3 Department of Health Law, Policy and Management, Boston University School of Public Health, Boston, MA USA; 4 Grayken Center for Addiction, Clinical Addiction Research and Education (CARE) Unit, Section of General Internal Medicine, Department of Medicine, School of Medicine, Boston Medical Center, Boston University, Boston, MA USA

**Keywords:** Traumatic brain injury, Opioid use disorder, Substance use disorder, Peer recovery support services, Latinx/Hispanic

## Abstract

**Background:**

Traumatic brain injury (TBI) is common in people with substance use disorders (SUDs). TBI often results in cognitive deficits which can affect the clinical course of SUD.

**Case presentation:**

Here we present the case of a 34-year-old Spanish-speaking man with severe opioid use disorder and two prior TBIs affecting his cognitive abilities. He was linked to outpatient addiction specialty care at a community health center. After identification of his TBI history, his care team, which included a language-concordant physician and peer recovery coach, worked to develop a treatment plan that accounted for his unique cognitive deficits and behavioral challenges. He was also connected with community resources including a rehabilitation program designed for people with TBI. These individualized aspects of treatment helped to better engage and retain the patient in quality care for his SUD.

**Conclusions:**

By identifying TBI history in people with SUDs, the treatment plan can be tailored to accommodate TBI-related deficits. An effective care plan should incorporate not only medical providers, but also resources such as peer recovery supports and TBI-focused rehabilitation programs when and where they are available, with an emphasis on improving functional capacity.

## Background

Traumatic brain injury (TBI), defined as an external traumatic force that alters normal brain function, is common in the United States. The CDC estimates that in 2019, there were more than 220,000 hospitalizations related to TBI and in 2021, there were almost 70,000 TBI-related deaths; this does not include the likely high numbers of unreported or untreated TBIs [1]. People who use substances are at increased risk for TBI and people with TBI are at increased risk for developing substance use disorders (SUD) [2]. There is growing evidence to suggest that childhood TBI increases risk for late adolescent and early adult substance use problems [3–5], and that TBI increases risk for prescription opioid receipt, opioid misuse, non-fatal overdose, and opioid use disorder (OUD) [6–8]. TBI likely complicates the treatment of SUD and vice versa, yet there has been little examination into these relationships. People with TBI who also have SUD may face additional challenges successfully engaging in SUD treatment because of neurobehavioral deficits common after TBI, and therefore would benefit from accommodations to make SUD treatment more cognitively accessible [6, 9]. Tailored interventions are needed to address the unique challenges faced by patients with these co-occurring conditions.

Here we present the case of a patient with a history of multiple TBIs affecting his cognitive abilities and subsequent development and treatment of opioid use disorder. We follow this case report with commentary from a TBI and addiction researcher as well as an experienced recovery coach who has worked with this patient and others facing similar obstacles. We conclude with lessons learned and implications for providers seeking to optimize care for patients with concomitant TBI and SUD.

## Case presentation

Mr. R is a 34-year-old monolingual Spanish-speaking man with severe OUD, prescribed buprenorphine-naloxone 8-2 mg three times a day by an outpatient provider. He was dissatisfied with his care and presented to a recovery coach (HL), fluent in Spanish, through a hospital-based substance use care navigation program. Recovery coaches provide emotional and social support and strategies for people with SUDs often built upon their own lived experience. In Massachusetts, recovery coach certification requires 500 hours of supervised volunteer or work experience, 60 hours of education across 8 domains over 10 years, and signing a code of ethics [10]. The recovery coach scheduled an appointment for Mr. R to establish care with an addiction medicine fellow physician (GR), also fluent in Spanish, in her continuity clinic at a community health center that provides integrated primary and substance use care.

For his first physician appointment, Mr. R presented 30 min late, accompanied by his recovery coach. During this visit, the physician struggled to obtain a clear substance use or medical history due to Mr. R’s tangential responses to questions. His primary substance use was intranasal use of fentanyl, with rare fentanyl smoking, as well as intermittent use of non-prescribed oxycodone-acetaminophen pills. He also smoked and used intranasal cocaine several times a month, which he stated was a decrease from previous daily use. He was abstinent from alcohol.

Regarding his opioid use, Mr. R said his use was triggered primarily by peers, including residents at his temporary housing unit, as well as frequent travel (including for medical visits) in areas of the city with concentrated open-air substance use. Despite his existing buprenorphine-naloxone prescription, Mr. R frequently forgot to take his medication as prescribed and continued to crave fentanyl. He had previously tried long-acting injectable (LAI) buprenorphine for two months, but stopped this due to local irritation at the injection site and because of ambivalence around his goals. The patient’s initial physical exam was notable for a depressed affect and a chronic contracture of his right hand due to congenital polio. He also exhibited issues with short-term memory and impulse control, which prompted further chart review and revealed the patient’s history of traumatic brain injury. The TBI history noted in the medical record then led to further discussion with Mr. R that elicited further details of his TBI as well as how it temporally related to his substance use. Below, we describe Mr. R’s TBI and substance use history, which was obtained over subsequent continuity clinic visits and through ongoing review of prior medical notes.

### Brain injury and substance use history

Mr. R was born and raised in El Salvador where he attended school through the fourth grade. At 11 years old, he fell off a horse, resulting in his first TBI. He lost consciousness for more than an hour, but did not seek medical care due to financial constraints. His first substance use was also around this time, when he began to drink alcohol. He then started using intranasal cocaine in his early teens. He immigrated to the United States when he was about 15 years old to join three sisters. In the United States, he attended an additional four months of ninth grade.

Mr. R continued to use alcohol and cocaine after immigrating to the United States, though neither he nor the medical record provided specific information on the quantity, frequency, or severity. He was diagnosed with a seizure disorder, which after brain imaging was attributed at least in part to neurocysticercosis. Ongoing seizures were triggered by episodes of cocaine use. Due to education regarding the connection between these seizures and alcohol and cocaine use, he decreased his cocaine use and stopped using alcohol during his later teen years. However, when he was 20 years old, he was hit by a car while on his bike and suffered from a second TBI with several hours of loss of consciousness, subsequently requiring bilateral craniotomies during a three-month hospitalization. He was using cocaine daily around this time, but reported that he was not intoxicated at the time of injury. He was admitted to the hospital and discharged on oral opioids for pain relief. This was his first time using opioids. Following this initial opioid prescription, he developed physical opioid dependence, which progressed to intranasal use of illicit opioids, and eventually an opioid use disorder.

In his twenties and early thirties, Mr. R had frequent acute medical visits for trauma and altered mental status, but no medical care continuity. He worked as a vendor in mobile food concession. During the year preceding his connection with the recovery coach and the physician, he had two overdoses leading to emergency department (ED) visits and reported additional overdoses that he could not quantify.

### Goals of care and challenges

Mr. R’s goals for care were to optimize his safety and increase control over his own substance use. His interest in stopping substance use altogether changed from one clinic visit to the next, fluctuating between ambivalence and a strong desire to stop. He also wanted to take care of his health by engaging more consistently with primary care. The physician, recovery coach, and patient identified challenges with transportation to clinic appointments and arriving on time for scheduled appointments, difficulty with medication adherence, struggles resisting peer pressure, inconsistent self-report of substance use (both due to memory deficits and reticence to disclose), and unstable housing.

### Accommodations and interventions by the care team and progression toward care goals

Over nine months of care, Mr. R’s clinical team developed and executed a treatment plan with specific accommodations for his identified needs. These included reminder calls for his scheduled appointment times and flexibility to be seen at some point during the half-day clinic session. The clinic offered fluent Spanish providers across the multidisciplinary care team. Because Mr. R struggled to remember the details of his substance use consistently, urine drug testing results were used to prompt his memory and discuss his strengths and triggers. His ongoing care was not contingent on these results. Mr. R’s recovery coach was invaluable in reminding and assisting him to attend scheduled clinic appointments and providing him with training for instrumental activities of daily living (IADLs), such as use of public transportation as well as use of a debit card. Given his difficulties with medication adherence, Mr. R was encouraged to again trial LAI buprenorphine. He agreed to this and received the LAI buprenorphine for three months, though subsequently opted to return to sublingual buprenorphine-naloxone films due to ongoing ambivalence around use.

Over time, Mr. R was better able to report drug use consistent with his urine drug screens. He obtained permanent stable housing, which helped him avoid triggering situations with people offering him drugs. Finally, he was linked to other community resources including food delivery service as well as a Spanish-speaking therapist through the community health center, and a Spanish-speaking primary care provider. He continued working with his recovery coach toward accessing services from a state-funded program designed to provide rehabilitation to individuals with a history of TBI. He had no known overdoses over the 12 months after engaging with regular medical care follow-up visits.

## Expert commentary

### Rachel Sayko Adams, PhD, MPH; Traumatic Brain Injury and Substance Use Disorder Researcher

1. *What features of this patient case are typical for people experiencing addiction who also have a history of TBI?*

There are many features of Mr. R’s presentation during addiction treatment that are consistent with cognitive impairments common following TBI and serve as a signal that he may have experienced a prior TBI. He was exhibiting issues with short-term memory and impulse control problems and was struggling to remember to take his addiction treatment medications. He had also been diagnosed with a seizure disorder; risk for seizures is elevated following more severe TBI [11]. Taken together, these cognitive impairments should trigger the clinician to screen for lifetime history of TBI, particularly when considering initiating SUD treatment. In Mr. R’s case, when cognitive impairments were identified in the first visit, this motivated the physician to review his chart, which revealed a prior TBI diagnosis and led to further assessment for lifetime history of TBI.

2. *Can you describe the 3-phase “perfect storm” model of cascading vulnerabilities for opioid use and consequences for people with a history of TBI? Does Mr. R’s case support the “perfect storm” model?*

Together with colleagues Drs. Corrigan and Dams-O’Connor [6, 7], we posited that there are cascading vulnerabilities for people with a history of TBI which make them uniquely vulnerable to consequences from opioid use, with each phase increasing risk for progression to the next. To summarize, Phase I states that people with TBI are at increased risk for receiving opioids largely due to disproportionate pain, other psychiatric comorbidities, and use of other substances at an earlier age. Phase II contends that once people with TBI start taking opioids that they are at greater risk for advancing to opioid misuse, long-term opioid therapy, or development of OUD. Phase III posits that if people with TBI develop OUD that they may face greater challenges accessing and engaging successfully in substance use disorder treatment due to neurobehavioral deficits or cognitive impairments following TBI, and a lack of appropriate accommodations. We noted that both Phases II and III of the model increase risk for devastating consequences such as overdose and death by suicide. To date, there has been a great deal of evidence to support Phases I and II of the “perfect storm” theory, with less known about Phase III in terms of potential disparities in access or outcomes for people with TBI engaging in substance use treatment, largely due to less research in this area. Emerging studies have found that people with TBI who are on long-term opioid therapy or develop substance use disorders are at increased risk for both non-fatal and fatal overdose, as well as death by suicide [12–16]. 

Mr. R’s case supports the “perfect storm” model in some important ways. While he began using other substances around the time of his childhood TBI, he reported that his first receipt of prescription opioids was following his second TBI which required hospitalization. Receipt of prescription opioids following this injury led to dependence and eventual development of OUD. Mr. R reported that he has experienced multiple non-fatal overdoses. When accessing substance use treatment, Mr. R had difficulties arriving on time for scheduled appointments, and self-reported use of substances inconsistently.

3. *What are the specific and special treatment needs of people with TBI and substance use?*

A recent study of US physicians found that only 41% reported feeling confident about their ability to provide the same quality care to patients with disabilities as those without disabilities [17]. Specific to TBI, studies have found that people with a TBI diagnosis were less likely to receive medications to treat OUD compared to people without a TBI diagnosis [18], and once initiating MOUD, they were less likely to continue MOUD [18, 19]. Differences in access and retention on MOUD may reflect healthcare inequities because there are no medical contraindications to using MOUD for people with TBI or other disabilities.

Implementing appropriate accommodations to address cognitive impairments following TBI can improve access and the likelihood of more successful substance use treatment outcomes, ultimately reducing risk for morbidity and mortality. Substance use treatment providers should be trained to accommodate executive functioning limitations and other cognitive impairments that are common following TBI when initiating and monitoring treatment, inclusive of MOUD [20, 21]. Examples include flexibility over missed visits, additional reminder calls, providing more opportunities for one-on-one interactions since group settings may be more difficult, and connection to a peer recovery coach. Many of these accommodations were introduced as a part of Mr. R’s treatment plan and appear to have been beneficial.

### Hansel Lugo, Recovery Coach/Patient Navigator for Mr. R

1. *How did your training and experience as a recovery coach prepare you to care for this patient?*

As a TBI survivor myself, and a person with lived substance use experience, I aim to treat others as I would have liked to be treated. That means I always treat each patient I care for with as much empathy and respect as I would want. I also hope to empower patients as partners in their personal treatment.

I have learned that you need to have a lot of patience when working with individuals who have suffered from a TBI. Regardless of its severity, a TBI can really affect someone’s temperament and sense of self. You have to understand that many were capable and had fulfilling lives before their injury. They sometimes feel like a part of them is lost because of their injuries and they feel like they are limited in living as they wish. It’s important to meet them where they are in their process of recovery.

2. *You work with many patients who do not speak English or only speak limited English. Are there additional considerations or special resources for these patients?*

Where I work in Boston, we have access to several community resources focused on Spanish-speaking individuals. Some examples include the East Boston Community Council, which has immigration services as well as High School Equivalency Test (HiSET) and General Education Development (GED) classes in Spanish, as well as La Colaborativa in Chelsea, which helps Latinx immigrant individuals and families address issues like housing insecurity, food insecurity, and education.

Unfortunately, I feel like there’s only a small number of social support programs geared toward Latinx and Hispanic individuals. The Latinx community really needs accessible resources like medications for SUDs, detoxification beds and services, and sober houses, and in particular services that are not limited based on insurance. For the programs in Boston that provide these services, space is limited and they cannot serve everyone. We really need to increase services that welcome the Latinx community.

## Lessons learned

1. Peer recovery coaches who are culturally responsive can engage and support patients with substance use disorders and multiple concomitant medical and mental health challenges.

Peer support services are a valuable element of the care plan for individuals with SUDs and have been a formalized aspect of care since the 1990s [22]. SAMHSA defines the support in this way: “a peer leader in stable recovery provides social support services to a peer who is seeking help in establishing or maintaining his or her recovery.” [23] There is a significant body of evidence showing the benefits of peer recovery social supports in SUDs. The first randomized controlled trial of a peer recovery support intervention showed those receiving peer support were more likely to abstain from cocaine at six-month follow-up [24]. The effectiveness of peer social supports has also been demonstrated specifically in individuals with OUD. Particularly relevant to Mr. R’s case is research that demonstrated an association between recovery coach contact and increased odds of buprenorphine treatment engagement and opioid abstinence [25]. 

For individuals like Mr. R from minoritized backgrounds, interaction with a peer recovery coach can address the cultural disconnect that is frequently a barrier to engaging with SUD treatment. One study conducted in low socioeconomic areas showed that implementation of a peer recovery training program that emphasized cultural responsiveness was associated with increased housing stability and employment for individuals with SUDs [26]. During his clinic visits, Mr. R consistently referenced the support he received from his peer recovery coach as crucial not only for his SUD recovery, but for his general wellbeing. This is consistent with qualitative research in which racial and ethnic minoritized participants more frequently referenced social support as a main driver in their engagement in addiction treatment [27].

2. Substance use care providers should have a high clinical suspicion for traumatic brain injury and consider screening and assessing.

Identification of TBI is an important first step to providing good care for individuals with TBI. This diagnosis enables both individuals and their care providers to understand possible associated deficits and to implement appropriate adaptations. TBI is frequently not an overtly apparent diagnosis and thus screening should be considered among populations at heightened risk [28]. TBI and SUD co-occur at high rates. TBI severe enough to cause loss of consciousness affects approximately 20% of Americans. One study found that 80% of those with co-occurring mental health and SUDs screened positive for TBI [29, 30]. In the case of Mr. R, his noted memory impairment and difficulty following procedural instructions prompted a more thorough chart review from GR, which revealed his TBI history. The Glasgow Coma Scale (GCS) is the most commonly used score to assess TBI severity [31]. Though this score was not available through chart review for Mr. R, his description of prolonged LOC as well as need for bilateral craniotomies suggests that he likely had a severe TBI. Implementing TBI screening into clinical practice could ensure that patients like Mr. R consistently receive the additional care supports that they need. The Massachusetts Rehabilitation Commission recommends considering TBI screening in four situations: during a new moment in care (e.g., a new patient appointment or a reassessment), when there is suspected trauma that could have caused a brain injury, when an individual is having difficulty functioning for unclear reasons, and/or when you suspect the individual has risk factors for TBI. We recommend that substance use care providers implement a brief screen for lifetime history of TBI for all new patients [20]. 

The Ohio State University TBI Identification Method (OSU TBI-ID) was developed based on the Centers for Disease Prevention and Control (CDC) definition of TBI and has been validated for use by anyone who has completed the tool’s free training [28, 32]. A shorter version of this is the OSU TBI-ID Quick Screen, which assesses any lifetime history of TBI with loss of consciousness, the severity of the TBI based on length of loss of consciousness, and the age at which the first TBI occurred. A positive screening questionnaire should prompt further assessment and creating a care plan.

3. Individuals with co-occurring substance use disorder and traumatic brain injury can be supported through individualized, tailored efforts aimed at specific cognitive deficits. Where and when available, local resources should be utilized for to support rehabilitation and maximizing function.

The fundamental pathophysiologic change seen in TBI is diffuse axonal injury (DAI) due to the strain and shearing injuries of brain nerve axons that occur with rapid acceleration and deceleration of the brain. Because of the anatomy of the brain, DAI tends to affect the frontal lobes and temporal lobes most significantly [33]. The frontal lobe is responsible for tasks of executive function, such as decision-making and problem-solving, while the temporal lobe is important for hearing, memory formation, and language recognition.

Mr. R’s situation serves as a case study of how this pathophysiology can present clinically and how his care team responded. For example, because Mr. R had difficulties with decision-making and had a particularly hard time saying no to an offer of drugs, one strategy was creating new routes for him to reach clinic in order to avoid passing certain triggering people or environments. Mr. R also faced challenges with memory, which affected him through difficulty taking his medications consistently and struggles to remember appointment times. In response to these concerns, his care team encouraged medications that required less frequent dosing when available or desirable (such as daily rather than twice a day medications and long-acting medication options) and also worked to provide him with a flexible appointment schedule. Finally, Mr. R’s sense of loneliness and isolation was typical of people with TBI that have diminished social activity levels and greater difficulty developing meaningful community connections [34]. To address this challenge, Mr. R’s care team encouraged him to engage in Spanish-speaking recovery support groups and to capitalize on his existing, though previously strained, connections with his sisters.

While this case focused on the impact of TBI and SUD for Mr. R, he, like other patients, had multiple likely contributors to his cognitive deficits, including prior nonfatal overdose, neurocysticercosis, and post-polio cognitive deficits. It is important to identify the other possible underlying etiologies of cognitive impairment in order to provide specific treatments, when there are any, and education about preventing recurrence and prognosis. However, the management strategies that we have highlighted that tailor care to the specific cognitive deficits should be relevant to patients struggling with cognitive impairment regardless of the specific mix of causes.

Additional in-person resources can be searched for and utilized depending on the local context. In Mr. R’s case, at the time of this case report, he was connected with the Massachusetts Rehabilitation Commission’s Statewide Head Injury Program (SHIP) [35]. This program offers services including adult companionship, skills training, and access to community centers. Unfortunately, because programs like SHIP are limited in number, not all patients in need will be able to consistently access their services. Research has also shown that individuals who, like Mr. R, are from racial and ethnic minority groups, are less likely to access rehabilitation or other posthospital care services after TBI. This has significant implications for long-term recovery from TBI [36, 37], and further demonstrates the importance of connecting patients from minoritized groups experiencing TBI and SUD with culturally appropriate peer support services.

## Conclusions

TBI and SUD are commonly co-occurring conditions that warrant specialized attention. The cognitive impairments that frequently develop following TBI have significant implications for effectively caring for people with SUDs. The first step to addressing co-occurring TBI and SUD is identification. Subsequently, to optimize SUD treatment for patients like Mr. R, a multifaceted and individualized care plan should be developed to address an individual’s specific needs regarding not only medical concerns but also daily functioning. Given the ongoing substance use and overdose epidemic, it is important to empower outpatient providers to make individualized accommodations to improve care for individuals with TBI and SUD. Furthermore, efforts should be made to expand the capacity and accessibility of local organizations that can offer expert support and interventions.

## Data Availability

No datasets were generated or analysed during the current study.

## Abbreviations

MVA: Motor vehicle accident
OBAT: Office based addiction treatment
OUD: Opioid use disorder
PRSS: Peer recovery support services
SUD: Substance use disorder
TBI: Traumatic brain injury
